# Experimental Study on a New Cement-Based Grouting Material for Iron Tailings Sand

**DOI:** 10.3390/ma19020328

**Published:** 2026-01-14

**Authors:** Ruibao Jin, Chaoyu Yang, Yangyang Luo, Yingchun Cai, Pan Guo, Dong Wei, Heng Liu

**Affiliations:** 1 Henan Transport Investment Jiaozheng Expressway Co., Ltd., Zhengzhou 450000, China; 2 School of Water Conservancy and Transportation, Zhengzhou University, Zhengzhou 450001, China; 3 Henan Province Highway Engineering Bureau Group Co., Ltd., Zhengzhou 450000, China; 4 School of Civil Engineering, Zhengzhou University, Zhengzhou 450001, China

**Keywords:** iron tailing sand, cementitious grout, replacement rate, flowability, compressive strength

## Abstract

This study develops a green, high-performance, cement-based grout by replacing manufactured sand with iron tailings sand (ITS) at ratios of 0–50% to address resource depletion. Fluidity, mechanical strength, and expansion rates were experimentally evaluated to determine engineering feasibility. The results indicate that while ITS inclusion reduces fluidity due to particle morphology, it significantly enhances compressive strength through a physical filling effect. Specifically, the 30% replacement group achieved a peak 28-day compressive strength of 100.4 MPa. Comprehensive analysis identifies 40% as the optimal replacement rate, where the grout strictly satisfies relevant industry specifications regarding fluidity, early strength, and volume stability. This research demonstrates the practical significance of utilizing industrial solid waste to produce high-performance sleeve grout for prefabricated construction.

## 1. Introduction

The building sector in China is expanding quickly, but as the demographic dividend disappears, labor is becoming more and more scarce, leading to a sharp rise in labor costs [1]. The fabricated building has been vigorously promoted and used in the construction industry due to its advantages of high construction efficiency, good economic efficiency, high safety, and low environmental pollution [2,3].

In assembled structures, node construction is a major problem. Reinforcement plays a significant part in the process of force transfer between members; thus, the connection of reinforcement is the primary node connection technique [4]. Grouted-sleeve connection is a type of mechanical connection technology for steel reinforcement, which is achieved by infusing early-strength, micro-expansion, high-strength, high-flow, cement-based grout [5,6]. Compared with other mechanical connection methods, it has the advantages of simple operation and easy construction, as well as low precision requirements in assembling, and has a wide range of application prospects [7]. The sleeve grout used for grouting sleeve connections is a type of cement-based grout. The working properties of grout have a great influence on the connection of grout sleeves [8]. Cement-based grout is a dry-mix material consisting of cement as the basic material, with additives, fine aggregates, and other materials. It has the properties of non-shrinkage, early strength, high strength, and large flowability after mixing with water, and is widely used in precast buildings [9,10].

Scholars have proposed various approaches to enhance cement-based grouting materials, primarily through additives and fiber reinforcement [11,12,13,14,15]. For instance, studies by Zhang et al. [16,17] demonstrated that incorporating fibers (e.g., basalt, PVA, and glass fibers) can effectively improve mechanical strength and toughness, though often at the cost of reduced fluidity. Liu et al. [18] prepared a new magnesium phosphate cement-based grouting material to study the effect of adding finely ground blast furnace slag (GGBS) on the performance of grouting materials. The results showed that GGBS could significantly shorten the setting time and increase fluidity to some extent. The addition of GGBS could improve the mechanical properties and water resistance of the grout. Shi et al. [19] conducted a feasibility study of alkali-resistant glass fiber (ARGF) as an additive to grout. The effects of the type and admixture amount of ARGF on the flexural strength, tensile strength, compressive strength, and seepage resistance of the grout were investigated. The results showed that ARGF can improve the compressive and tensile strength of grout, and grout with an ARGF volume fraction of 0.5% has the best mechanical properties. Myrsini et al. [20] investigated the electrical energy-thermal energy conversion capability of cementitious materials by adjusting the electrical conductivity and energy storage properties of carbon nanotubes (CNTs) and nanofibers (CNFs) to enhance cementitious materials based on the research basis. The results showed that the degree of dispersion of CNTs and CNFs, and high electrical and thermal conductivity values are essential to achieve electrical-to-thermal energy conversion; a low amount of uniformly dispersed CNTs and CNFs enhanced electrothermal conversion efficiency.

Iron ore tailings are a major component of industrial solid waste. Statistics show that China produces around 1 billion tons of tailings per year [21]. A total of 1.211 billion tons of tailings were produced in China in 2018 as a result of these processes, with iron tailings making up around 476 million tons, or 39.31%, of the total volume produced [22,23]. Iron tailings are generally disposed of by direct landfilling, but this causes both serious pollution to the surrounding ecological environment and waste of land resources. In addition, a large number of piles of iron tailings may also cause disaster accidents [24,25]. In the current situation of resource scarcity, the use of iron tailings as raw materials for construction materials can realize “turning waste into treasure”, and solve the problems of land resource occupation and environmental pollution [26].

In this study, high-performance grout is made using iron tailing sand in place of certain manufactured sand, and the iron tailing sand replacement rate is used as the variable for the design of the experimental scheme. Fluidity, flexural strength, compressive strength, and swelling rate tests were used to investigate the effect of the rate of iron tailing sand replacement on the mechanical characteristics and workability of iron tailing sand cement-based grout. The optimal replacement rate of iron tailings slag was determined by experimental comparison.

## 2. Experimental Program

### 2.1. Material Properties

Manufactured sand was provided by a commercial concrete company in Xingyang City, Zhengzhou City, with a bulk density of 1670 kg/m^3^ and a fineness modulus of 2.9. The grain size sieve residual analysis of the manufactured sand is shown in Table 1.

**Figure 1:**
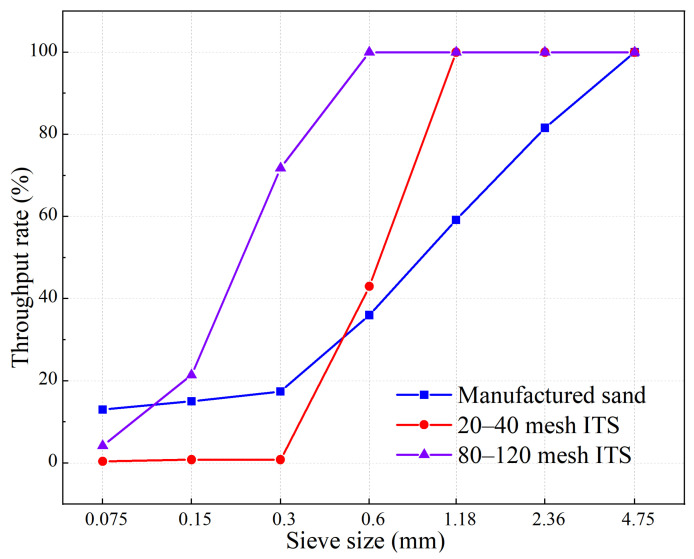
Grain size sieving rate of three types of sand.

Iron tailing sand from the Hebei area with a bulk density of 1703 kg/m^3^ (80–120 mesh) was used. As shown in Table 2, three groups of specimen sand were randomly selected before the test, and after passing through a 4.75 mm square hole sieve, the average value of fineness modulus of 80–120 mesh iron tailing sand was measured as 1.1, and the fineness modulus of 20–40 mesh iron tailing sand was 2.6. The physical properties of iron ore tailings are similar to those of natural sand [25], and the particle size sieving rates of the manufactured sand and iron tailings sand are shown in Figure 1. To verify the consistency of the raw materials, the particle size distribution and sieving rates were further analyzed. The sieving results indicate that the iron tailings sand possesses a continuous and stable gradation. Furthermore, the minimal deviation in fineness modulus across the randomly sampled groups verifies the high uniformity of the material. This consistent particle gradation ensures the reliability of the aggregate skeleton, minimizing experimental error caused by raw material variability.

The fineness of the aggregates has a large impact on the performance of cementitious grout. Shettima et al. [25] used iron tailings sand with a fineness modulus of 1.05 instead of fine aggregate to prepare concrete. Taking this into account, this study is proposed to be conducted with iron tailing sand of 1.1 fineness modulus.

Previous studies have shown that while the chemical composition of iron tailings varies significantly across different regions and mine types, SiO_2_ and Fe_2_O_3_ consistently remain the predominant constituents [27]. This commonality establishes a technical foundation for utilizing iron tailings as a sustainable construction material. The specific chemical properties of the iron tailings used in this study are detailed in Table 3. While the iron tailings used in this study exhibit low sulfur content and volume stability, compositional variability (e.g., pyrite) in other sources poses durability risks such as delayed ettringite formation. Therefore, pretreatment-like desulfurization is essential for high-sulfur tailings in practical engineering to meet standard limits.

### 2.2. Preparation

The mix proportions were determined based on extensive preliminary experiments and relevant literature [25,26]. First, the water-to-binder ratio and admixture dosage were optimized through trial mixes to ensure the fluidity met the requirements of JG/T 408-2019. Subsequently, a mass replacement method was adopted, where iron tailings sand replaced manufactured sand at ratios of 10%, 20%, 30%, 40%, and 50%, a range selected based on previous findings that balanced solid waste utilization with mechanical performance [27]. The specific mix proportions for each group are detailed in Table 4. The preparation process of the grout is as follows: first, 1800 g of cement-based grout and 171 g of water were weighed according to the mix proportion of each group. The mixing pot and mixing blades were wetted and then wiped with a damp cloth. The dry mixture was poured into the mixing pot, the mixer was activated, and the mixing water was added simultaneously within 10 s. The mixing program of the cement mortar mixer was set to 240 s, consisting of 90 s of low-speed mixing, 70 s of rest, and 80 s of high-speed mixing. The mixture was allowed to stand for 2 min after mixing was completed to discharge any internal air bubbles.

### 2.3. Specification Requirements

Unlike ordinary mortar, sleeve grout for assembly construction should have good fluidity to ensure the denseness of the grout, as well as high compressive strength and expansion to ensure the reliability of the grouted sleeve joints. Sleeve Grout for Steel Sleeve Connection (JG/T 408-2019) [28] is an important standard for testing whether iron tailing sand cement-based grout meets the requirements of sleeve grout, and in accordance with the specification, sleeve grout must meet the relevant requirements of Table 5.

### 2.4. Test Scheme

#### 2.4.1. Fluidity Test

The fluidity of the grout was evaluated in strict accordance with sleeve grout for reinforcing steel connections [28]. The testing apparatus consisted of a glass plate (500 mm × 500 mm) and a truncated cone mold (top inner diameter 70 mm, bottom inner diameter 100 mm, height 60 mm). Prior to testing, the inner wall of the mold and the glass plate were moistened with water, ensuring no standing water remained. The glass plate was placed horizontally, and the mold was centered on the plate. The grout was poured into the mold until it was flush with the top edge. The mold was then lifted vertically, allowing the grout to flow freely. The spread diameter was measured in two perpendicular directions using a ruler, and the average value was recorded as the initial fluidity. For the 30 min fluidity retention test, the mixture was kept in the mixing pot and covered with a damp cloth to prevent moisture evaporation before repeating the measurement steps.

#### 2.4.2. Flexural Strength Test

Flexural strength was determined using prism specimens with dimensions of 40 mm × 40 mm × 160 mm, following the protocols in JG/T 408-2019 and Method of Testing Cements—Determination of Strength (GB/T 17671-1999) [29]. The grout was poured into the molds without vibration to ensure natural formation, leveled, and immediately covered with plastic film. The specimens were initially cured in a molding room for 2 h, then transferred to a curing box for 22 h before demolding. Subsequently, the specimens were cured in a standard curing room (temperature 20 ± 3 °C, relative humidity ≥ 95%) until the designated testing age. A flexural testing machine was used to apply a load to the side faces of the prism at a rate of 50 N/s until fracture. The final flexural strength was the average of three specimens.

#### 2.4.3. Compressive Strength Test

Following the flexural test, the six broken halves of the prism specimens were used for the compressive strength test. The specimens were placed in a hydraulic pressure testing machine using the smooth side faces (40 mm × 40 mm) as the bearing surface. The load was applied uniformly at a rate of 2.4 kN/s until failure. The compressive strength was calculated as the average of the six measurements. If any individual result deviated by more than 10% from the mean, the entire group was discarded and retested.

#### 2.4.4. Vertical Expansion Rate Test

The vertical expansion rate was measured using a steel mold (100 mm × 100 mm × 100 mm) equipped with a dial gauge, as specified in JG/T 408-2019. The grout was poured into the mold from one side until the level on the opposite side was approximately 2 mm above the mold edge. Slight manual vibration was applied during pouring to ensure compaction. After pouring, the exposed surface was covered with a wet cotton cloth. Height readings were recorded at 3 h (h_0_) and 24 h (h_t_). The vertical expansion rate (ε_t_) was calculated using Equation (1):

(1) $$\varepsilon_{t}=\frac{h_{t}-h_{0}}{h}\times 100\%$$

where ε_t_ is the vertical expansion rate (%); h_0_ is the initial height reading (mm); h_t_ is the height reading at age t (mm); and h is the reference height of the specimen (taken as 100 mm).

#### 2.4.5. Statistical Analysis

All experimental data are presented as the mean ± standard deviation. A one-way analysis of variance (ANOVA) was performed to evaluate the statistical significance of the differences in mechanical properties, with a *p*-value of less than 0.05 (*p* < 0.05) considered statistically significant.

## 3. Test Results and Analysis

### 3.1. Fluidity

**Figure 2:**
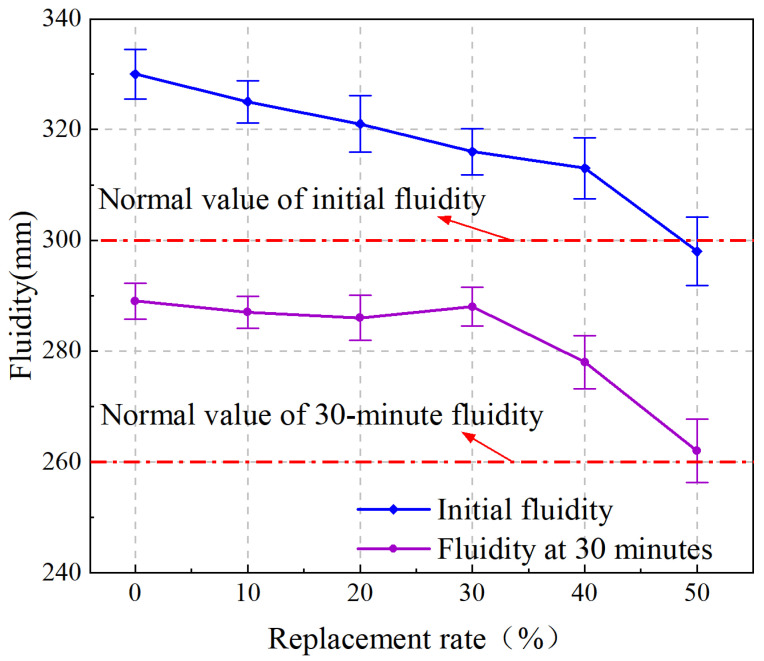
Variation curve of fluidity with iron tailing sand replacement rate.

The variation curve of the fluidity of iron tailings sand cement-based grout with iron tailings sand substitution rate is shown in Figure 2. As the replacement rate of iron tailing sand increased, its initial value of flowability, as well as the retention value after 30 min, decreased, indicating that the admixture of iron tailing sand was unfavorable to the flowability of cementitious grout, but the water retention of each group of iron tailing sand cementitious grout and the baseline group of the grout was good and no water secretion occurred.

The use of iron tailing sand with a small fineness modulus to replace a portion of the larger fineness modulus of the manufactured sand leads to a decrease in the flow of the grout for a number of reasons. Firstly, iron tailings sands consist of irregularly shaped, porous particles [30], and the irregular shape combined with loose particles leads to high surface area and greater water demand [25], which results in reduced flowability. Secondly, compared with ordinary sand, the surface of iron tailing sand particles is rough, which contains more needle-like particles, and the plastic viscosity of iron tailing sand cement-based grout is higher, and its construction performance is slightly worse than that of ordinary grout. This conclusion is further supported by Shettima et al. [25], who confirmed that, beyond water demand, the high angularity and surface roughness of tailings significantly increase inter-particle friction compared to conventional manufactured sand, thereby reducing the overall workability of the mix. It is important to emphasize that fluidity is a mandatory performance index for sleeve grouting materials as stipulated by the Chinese standard Sleeve Grout for Reinforcing Steel Connections (JG/T 408-2019) [29]. This standard imposes strict requirements on both initial fluidity (≥300 mm) and 30 min fluidity retention (≥260 mm) to guarantee the filling quality of the sleeve. Therefore, this study prioritizes comparing the experimental results with these standard limits to evaluate the engineering applicability of the proposed material.

**Figure 3:**
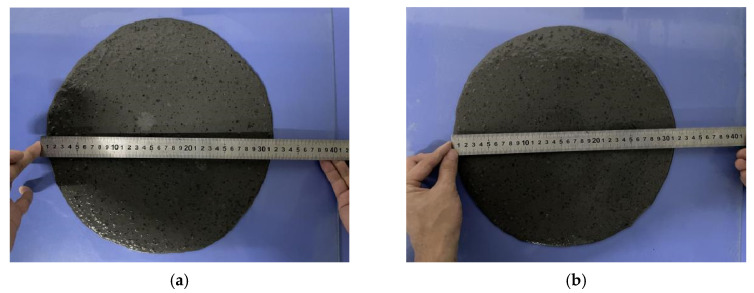
Fluidity test process: (**a**) representative photo of the control group (the reported 330 mm is the average of 3 trials); (**b**) representative photo of the 40% replacement group.

The substitution of iron tailing sand for manufactured sand reduces the fluidity of cementitious grout. When the content of iron tailing sand in cementitious grout was increased to 50%, the flow rate decreased by 9.7% compared to the control group. Ordinary manufactured sand grout is more workable than grout containing iron tailing sand. When the iron tailing sand replaces 40% of the manufactured sand admixture, it meets the specification requirements. When the replacement rate of iron tailing sand reaches 50% admixture, the mix shows larger viscosity and poor fluidity; its 30 min flow rate meets the requirements, and the initial flow rate is small and cannot meet the specification requirements. The initial flow tests for the control and 40% groups are shown in Figure 3.

### 3.2. Compressive Strength

**Figure 4:**
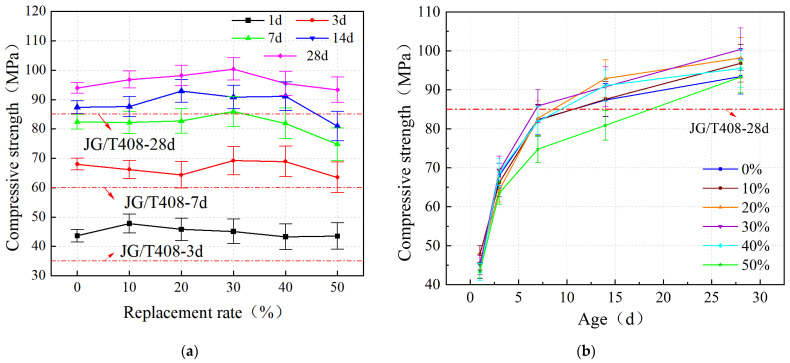
Compressive strength of grout: (**a**) variation with replacement rate; (**b**) variation with age.

The effect of iron tailing sand on the compressive strength of the grout at different ages is shown in Figure 4a. With the increase in iron tailing sand replacement rate, its strength has an undulating trend, showing an overall trend of increasing and then decreasing, and the greater the age, the greater the dispersion. The compressive strength laws at 1 d, 7 d, 14 d, and 28 d ages are obvious, showing an overall trend of first growth and then decrease. At the age of 1 d, the compressive strength peaked at 10% replacement rate, at 47.8 MPa, which was 9.6% higher than the control group. At the age of 3 d, the compressive strength peaked at 30% substitution rate at 69.2 MPa, which was 1.8% higher than the control group. At the age of 7 d, the compressive strength peaked at 30% replacement rate at 85.9 MPa, which was 4.2% higher than the control group. At 14 d of age, the compressive strength peaked at 20% substitution rate at 92.9 MPa, which was 6.3% higher than the control group. At the age of 28 d, the compressive strength peaked at 30% substitution at 100.4 MPa, which was 6.9% higher than the control group.

When the replacement rate was 50%, the compressive strength at all ages was smaller than that of the control group, indicating that when the replacement rate of iron tailing sand was greater than 50%, the compressive strength showed a decreasing trend, at which time iron tailing sand could no longer improve the compressive strength of the grout.

All experimental data are presented as the mean ± standard deviation. The results of the one-way ANOVA indicated that the replacement rate had a statistically significant effect on the compressive strength (*p* < 0.05). Specifically, compared with the control group, the strength improvement observed in the 20% and 30% replacement groups was significant, confirming the positive reinforcing effect of iron tailings sand at the appropriate replacement ratios dosage.

As shown in Figure 4b, the compressive strength of iron tailings sand cement-based grout with different replacement rates showed a similar growth trend with age, and the strength growth was faster at 1–7 days. The compressive strength of each group of grout reached about 50% of the 28 d strength specification value of 85 MPa at the age of 1 d. At the age of 3 d, the compressive strength reached about 75% of the 28 d strength specification value. When the replacement rate is 30%, the compressive strength at 7 d age reaches 85.9 MPa, which has reached the required value of 28 d strength specification. It shows that when the iron tailing sand replacement rate is less than 50%, the grout exhibits early strength performance.

When the age reached 14 d, all groups with less than 50% replacement rate had exceeded the 28 d specification strength value of 85 MPa, except for the compressive strength of the 50% iron tailing sand admixture group, which did not reach the 28 d strength specification requirement. The development of compressive strength slowed down significantly during the age period of 7–14 d. The development of compressive strength at the age of 14–28 d was more moderate compared to the age of 7–14 d, but still increased. It shows that the strength of iron tailing sand cement-based grout has stabilized at 14 d. It can also be seen that the greater the age, the longer the specimen is maintained, and the greater the dispersion.

**Figure 5:**
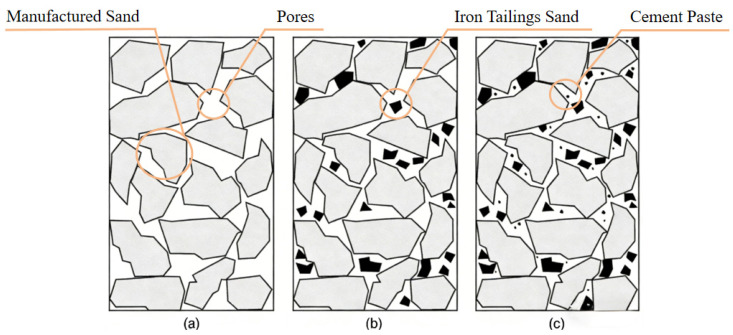
(**a**) Manufactured sand (polygons) + pores; (**b**) Manufactured sand (polygons) + iron tailings sand (medium circles); (**c**) Full system: manufactured sand (polygons), iron tailings sand (medium circles), cement paste (small particles).

The mechanism behind the strength enhancement is primarily physical rather than chemical. Since the iron tailings sand (ITS) used in this study (fineness modulus 1.1) is significantly finer than the manufactured sand (fineness modulus 2.9), it acts as a micro-filler, effectively occupying the voids between the coarser aggregate particles. This improves the packing density of the granular skeleton, as schematically illustrated in Figure 5. Additionally, the irregular and angular texture of ITS particles enhances the mechanical interlocking force with the cement paste, further resisting compressive stress. This physical densification effect aligns with the findings of Shettima et al. [27], who observed that appropriate tailings inclusion reduces porosity and increases density. Furthermore, recent studies have highlighted that specific micro-structural characteristics, such as the distribution of pore length and volume, play a governing role in the mechanical functionality of cementitious materials [31]. Quantitatively, the compressive strength obtained in this study (peaking at 100.4 MPa) is consistent with the order of magnitude reported by Zhao et al. [27] for similar iron tailings-based high-performance composites [32], validating that the physical filling effect is the dominant factor at this replacement level.

### 3.3. Flexural Strength

**Figure 6:**
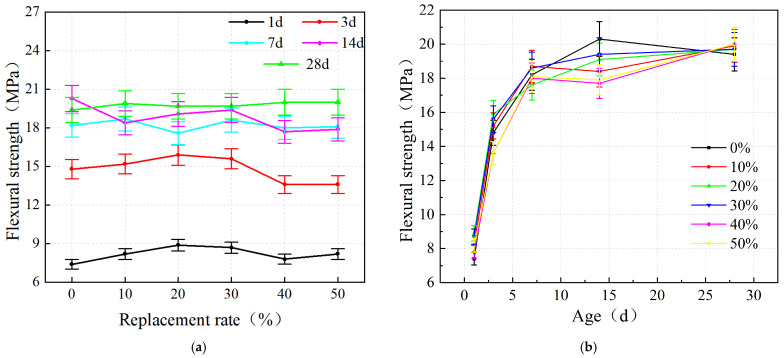
Flexural strength results: (**a**) variation with replacement rate; (**b**) variation with age.

The effect of iron tailing sand on the flexural strength of the grout at different ages is shown in Figure 6a. As the replacement rate of iron tailing sand increases, its strength also tends to rise and fall, again showing an overall trend of first increasing and then decreasing. The flexural strength increases with increasing substitution rate at substitution rates of 0 to 20% in 1 d and 3 d ages. When the substitution rate exceeds 30%, the flexural strength decreases with increasing substitution rate. At 1 d of age, the flexural strength peaked at 20% replacement rate at 8.9 MPa, which was 20% higher than the control group. At the age of 3 d, the flexural strength peaked at a substitution rate of 20% at 15.9 MPa, which was 7.4% higher than that of the control group. The peak flexural strengths of the iron tailing sand cement-based grout at 7 d, 14 d, and 28 d ages were 18.7 MPa, 20.3 MPa, and 20 MPa, respectively, with a small difference between the three. It indicates that the flexural strength almost reaches its peak at 7 d. Thereafter, there is no significant enhancement of flexural strength with the increase in age. Meanwhile, the strength difference between 7 d and 3 d is significantly smaller than that between 3 d and 1 d, indicating that the flexural strength develops faster and stabilizes earlier than the compressive strength.

Statistical analysis confirmed significant differences in flexural strength among the groups (*p* < 0.05). The peak flexural strength observed at the 20% replacement rate was significantly higher than that of the control group, suggesting that the interlocking effect of the angular iron tailings particles effectively enhanced the bending resistance.

As shown in Figure 6b, flexural strength development shows a similar growth trend with age. At the early 1–7 d age, flexural strength develops faster. At 7 d of age, the compressive strength has reached about 92% of 28 d age. The strength development tends to be stable from 7 to 14 d. Compared with the compressive strength, the flexural strength reaches the peak strength earlier, which also reflects the early strength mechanical properties of the grout. The flexural strength hardly develops anymore or even decreases during 14–28 d.

### 3.4. Vertical Expansion Rate

**Figure 7:**
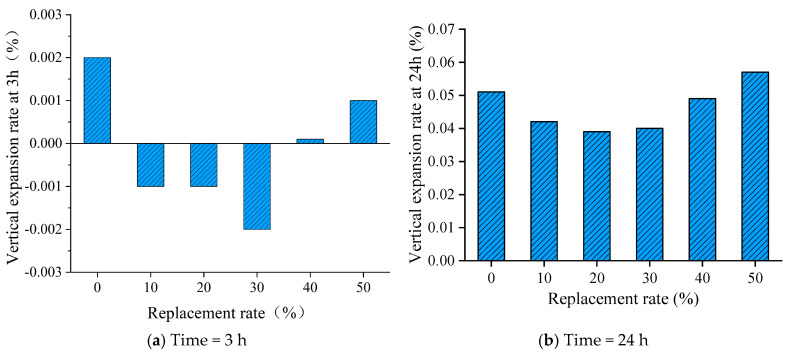
Vertical expansion rate with time.

As shown in Figure 7a, the 10%, 20%, and 30% iron tailings sand cementitious grout groups showed minimal shrinkage within 3 h. In contrast, the 3 h vertical expansion of the all-manufactured sand grout and the 40% and 50% iron tailing sand cement-based grout groups, which were used as the reference groups, were almost zero. This indicates that the expander is not yet effective within 3 h of grout forming. As shown in Figure 7b, the vertical expansion rate showed a gradual increase with time, and it showed a trend of decreasing and then increasing with the increase of iron tailing sand replacement rate. The minimum 24 h vertical swelling rate was 0.039 at 20% iron tailing sand replacement rate, which was 23.5% lower than the swelling rate of the control group. The maximum 24 h vertical swelling rate was 0.057 at 50% iron tailing sand replacement rate; relative to the control group swelling rate, it was increased by 11.8%. Although the irregular morphology of iron tailings sand leads to a slight decrease in fluidity compared to manufactured sand, the workability and expansion rates at the 40% replacement rate still strictly meet the standard requirements of sleeve grout for steel connection (JG/T 408-2019) [29]. Furthermore, the appropriate incorporation of iron tailings sand effectively improves the mechanical properties, particularly the compressive strength. Therefore, the proposed mix design successfully achieves a balance between utilizing solid waste and maintaining high mechanical performance.

## 4. Conclusions

In this paper, iron tailings sand is used to replace manufactured sand to prepare a green and environmentally friendly new iron tailings sand cement-based grouting material. The effects of five different dosages on the fluidity, compressive and flexural strength, vertical expansion rate, and the flexural-compressive ratio of grouting material are studied and analyzed. The conclusions are as follows:

(1) The fluidity of cement-based grouting material is decreased when manufactured sand is replaced with iron tailings sand. It complies with the specifications when the iron tailings sand substitutes 40% of the produced sand. The initial fluidity is low and falls short of the specifications when iron tailings sand substitutes manufactured sand to a 50% ratio.
(2) With the increase in the replacement rate of iron tailings sand, the compressive strength generally shows a trend of increasing first and then decreasing, and the replacement rate reaches a peak of 100.4 MPa at 30%. The strength rose significantly between the ages of 0–7 d, reaching the lowest value of 85 MPa in the specified 28 days at 14 days, which reflected the characteristics of early strength.
(3) With the increase of iron tailings sand replacement rate, the flexural strength at 1 d and 3 d ages showed a trend of first increase and then decrease, and reached the peak at 20% with 8.9 MPa and 15.9 MPa, respectively. The changes in flexural strength at 7 d, 14 d, and 28 d age groups were small, indicating that the effect of iron tailings sand replacement rate on flexural strength was relatively small, but still showed a general trend of first increase and then decrease.
(4) The 3 h vertical expansion of the iron tailing sand cementitious grout was almost zero at each substitution rate. The 24 h vertical expansion rate of each replacement rate of iron tailing sand cement-based grout meets the relevant specification requirements. The 24 h vertical expansion rate of grout shows a trend of decreasing and then increasing as the replacement rate of iron tailing sand increases.
(5) Considering the flow rate, flexural and compressive strength, and relevant specifications, the optimal replacement rate is 40% when iron tailings sand is substituted for replacement manufactured sand. However, this study was limited to the use of iron tailings sand with a fineness modulus of 1.1. Since aggregates with different particle sizes and surface roughness characteristics can have distinct effects on the performance of cement-based grouting materials, further research is needed to investigate the basic mechanical properties of grouting materials prepared with iron tailings sand of varying particle sizes.

## Figures and Tables

**Table 1:** Manufactured sand sieve residual analysis.

Sieve Residue/%	Grain Size/mm								Fineness Modulus
	Sieve Bottom	0.075	0.15	0.3	0.6	1.18	2.36	4.75	
Subtotal	15.0	2.0	2.4	18.6	23.2	22.4	18.4	0	2.9
Cumulative	100.0	87.0	85.0	82.6	64.0	40.8	18.4	0	

**Table 2:** Iron tailing sand sieve residual analysis.

Category	Sieve Residue/%	Grain Size/mm								Fineness Modulus
		Sieve Bottom	0.075	0.15	0.3	0.6	1.18	2.36	4.75	
20–40 mesh	Subtotal	0.4	0.4	0	42.2	57.0	0	0	0	2.6
	Cumulative	100	99.6	99.2	99.2	57.0	0	0	0	
80–120 mesh	Subtotal	3.4	14.6	53.4	28.6	0	0	0	0	1.1
	Cumulative	100	96.6	82.0	28.6	0	0	0	0	

**Table 3:** Chemical composition of iron ore tailing sand.

SiO_2_	Fe_2_O_3_	MgO	CaO
42%	25%	3%	6%

**Table 4:** Mix proportion.

Content of Iron Tailing Sand	0%	10%	20%	30%	40%	50%
Cement (g)	810	810	810	810	810	810
Mineral dopants (g)	162	162	162	162	162	162
Additives (g)	18	18	18	18	18	18
Manufactured sand (g)	810	729	648	567	486	405
Iron tailing sand (g)	0	81	162	243	324	405
Water (g)	171	171	171	171	171	171

**Table 5:** Technical performance index of sleeve grout.

Fluidity (mm)		Compressive Strength (MPa)			Vertical Expansion Rate (%)		Chlorine Ion Content (%)	Bleeding Rate (%)
Initial	30 min	1 d	3 d	28 d	3 h	Difference between 24 h and 3 h	≤0.03	0
≥300	≥260	≥35	≥60	≥85	≥0.02	0.02~0.5		

## Data Availability

The original contributions presented in this study are included in the article. Further inquiries can be directed to the corresponding author.

## References

[1] McNeil M.A., Feng W., De La Rue Du Can S., Khanna N.Z., Ke J., Zhou N. (2016). Energy efficiency outlook in China’s urban buildings sector through 2030. Energy Policy.

[2] Shufeng L., Qingning L., Hao Z., Haotian J., Lei Y., Weishan J. (2018). Experimental study of a fabricated confined concrete beam-to-column connection with end-plates. Constr. Build. Mater..

[3] Deng E.-F., Zhang Z., Zhang C.-X., Tang Y., Wang W., Du Z.-J., Gao J.-P. (2023). Experimental study on flexural behavior of UHPC wet joint in prefabricated multi-girder bridge. Eng. Struct..

[4] Zheng G., Kuang Z., Xiao J., Pan Z. (2020). Mechanical performance for defective and repaired grouted sleeve connections under uniaxial and cyclic loadings. Constr. Build. Mater..

[5] Ling J.H., Rahman A.B.A., Ibrahim I.S., Hamid Z.A. (2016). Tensile capacity of grouted splice sleeves. Eng. Struct..

[6] Liu C., Pan L., Liu H., Tong H., Yang Y., Chen W. (2020). Experimental and numerical investigation on mechanical properties of grouted-sleeve splices. Constr. Build. Mater..

[7] Lu Z., Wu B., Yang S., Hou J., Ji Z., Li Y., Huang J., Zhang M. (2021). Experimental study on flexural behaviour of prefabricated concrete beams with double-grouted sleeves. Eng. Struct..

[8] Liu H., Chen J., Xu C., Du X. (2020). Seismic performance of precast column connected with grouted sleeve connectors. J. Build. Eng..

[9] Dai Z., Cheong T.Y.C., Pang S.D., Liew J.Y.R. (2021). Experimental study of grouted sleeve connections under bending for steel modular buildings. Eng. Struct..

[10] Kuang Z., Zheng G. (2018). Computational and Experimental Mechanical Modelling of a Composite Grouted Splice Sleeve Connector System. Materials.

[11] Lei S., Liu L., Wu F., Lin W., Peng K. (2023). Experimental study on tensile mechanism of UHPC grouted sleeve splice. Constr. Build. Mater..

[12] Wang J., Zhang H., Zhu Y., Yan Z., Chai H. (2022). Acrylamide in-situ polymerization of toughened sulphoaluminate cement-based grouting materials. Constr. Build. Mater..

[13] Zhang H., Yao S., Wang J., Zhou R., Zhu Y., Zou D. (2023). Tensile properties of sulfoaluminate cement-based grouting materials toughened by in-situ polymerization of acrylamide. Constr. Build. Mater..

[14] Zhang C., Shuai B., Jia S., Lv X., Yang T., Chen T., Yang Z. (2021). Plasma-functionalized graphene fiber reinforced sulphoaluminate cement-based grouting materials. Ceram. Int..

[15] Zhao W., Feng S., Liu J., Sun B. (2023). An explainable intelligent algorithm for the multiple performance prediction of cement-based grouting materials. Constr. Build. Mater..

[16] Zhang P., Su Y.L., Fan J.J., Feng H., Shao J., Guo H., Sheikh S.A. (2021). Experimental research on the mechanical behavior of grouted sleeves with fiber-reinforced grouting material under cyclic loading. Structures.

[17] Zhang P., Yu J., Pang Y., Fan J., Guo H., Pan Z. (2021). Experimental study on the mechanical properties of grouted sleeve joint with the fiber-reinforced grouting material. J. Build. Eng..

[18] Liu Y., Chen B. (2019). Research on the preparation and properties of a novel grouting material based on magnesium phosphate cement. Constr. Build. Mater..

[19] Shi Z., Wang Q., Xu L. (2020). Experimental Study of Cement Alkali-Resistant Glass Fiber (C-ARGF) Grouting Material. Materials.

[20] Maglogianni M.E., Danoglidis P.A., Konsta-Gdoutos M.S. (2023). Electrical-to-thermal energy conversion efficiency of conductive concrete. Cem. Concr. Compos..

[21] Zhang Z., Zhang Z., Yin S., Yu L. (2020). Utilization of Iron Tailings Sand as an Environmentally Friendly Alternative to Natural River Sand in High-Strength Concrete: Shrinkage Characterization and Mitigation Strategies. Materials.

[22] Zhu Q., Yuan Y., Chen J., Fan L., Yang H. (2022). Research on the high-temperature resistance of recycled aggregate concrete with iron tailing sand. Constr. Build. Mater..

[23] Lai Y., Yu J., Liu S., Liu J., Wang R., Dong B. (2021). Experimental study to improve the mechanical properties of iron tailings sand by using MICP at low pH. Constr. Build. Mater..

[24] Huang X., Ranade R., Ni W., Li V.C. (2013). Development of green engineered cementitious composites using iron ore tailings as aggregates. Constr. Build. Mater..

[25] Shettima A.U., Hussin M.W., Ahmad Y., Mirza J. (2016). Evaluation of iron ore tailings as replacement for fine aggregate in concrete. Constr. Build. Mater..

[26] Zhao Y., Gu X., Qiu J., Zhang W., Li X. (2021). Study on the Utilization of Iron Tailings in Ultra-High-Performance Concrete: Fresh Properties and Compressive Behaviors. Materials.

[27] Zhao J., Ni K., Su Y., Shi Y. (2021). An evaluation of iron ore tailings characteristics and iron ore tailings concrete properties. Constr. Build. Mater..

[28] (2019). Sleeve Grout for Reinforcing Steel Connections.

[29] (1999). Method of Testing Cements—Determination of Strength.

[30] Zhao S., Fan J., Sun W. (2014). Utilization of iron ore tailings as fine aggregate in ultra-high performance concrete. Constr. Build. Mater..

[31] Salha M.S., Adenusi H., Setiadi D.H., Yada R.Y., Farrar D.H., Di Tommaso D., Tian K.V., Chass G.A. (2026). Al-driven cement functionality by manifold structuring & disorder. Mater. Adv..

[32] Liu G.L., Zhao P., Qin L., Ren H.W. (2021). Composition and Analysis of Domestic Patented Sleeve Grouting Material. Mater. Sci. Forum.

